# Professor Hamilton’s Translation of M. Amussat’s Treatise

**Published:** 1851-09

**Authors:** 


*Professor Hamilton's translation of M. Amussat's treatise.*—This excellent translation, published in successive numbers of this journal, has been issued separately in a very neat volume, which we received just as we were completing the matter for our editorial department. It has been very favorably noticed by the medical press. We have only space in this No. for the following from the N. Y. Med. Gazette:

*Water in Surgery.*—Professor Hamilton, of Buffalo, has done good service to the profession and the public, by the English dress in which he has published M. Amussat's thesis on the value of water as a therapeutic agent. It it could be extensively circulated, the cunning wights who have stolen the thunder of the regular profession to gull the public into a belief that there is any novelty in their Hydropathy which is true, would find "Othello's occupation gone."

